# Risk Recognition, Attachment Anxiety, Self-Efficacy, and State Dissociation Predict Revictimization

**DOI:** 10.1371/journal.pone.0108206

**Published:** 2014-09-19

**Authors:** Estelle Bockers, Stefan Roepke, Lars Michael, Babette Renneberg, Christine Knaevelsrud

**Affiliations:** 1 Department of Clinical Psychology and Psychotherapy, Freie Universität Berlin, Berlin, Germany; 2 The Berlin Center for Torture Victims, Berlin, Germany; 3 Department of Psychiatry, Charité – Universitätsmedizin Berlin, Campus Benjamin Franklin, Berlin, Germany; 4 Cluster of Excellence “Languages of Emotion”, Freie Universität Berlin, Berlin, Germany; 5 Medical School Hamburg, University of Applied Sciences and Medical University, Hamburg, Germany; Queen Mary University of London, United Kingdom

## Abstract

**Background:**

Previous research has identified a number of variables that constitute potential risk factors for victimization and revictimization. However, it remains unclear which factors are associated not only with childhood or adolescent victimization, but specifically with revictimization. The aim of this study was to determine whether risk recognition ability and other variables previously associated with revictimization are specifically able to differentiate individuals with childhood victimization only from revictimized individuals, and thus to predict revictimization.

**Methods:**

Participants were *N* = 85 women aged 21 to 64 years who were interpersonally victimized in childhood or adolescence only, interpersonally revictimized in another period of life, or not victimized. A logistic regression analysis was conducted to examine whether risk recognition ability, sensation seeking, self-efficacy, state dissociation, shame, guilt, assertiveness, and attachment anxiety predicted group membership.

**Results:**

The logistic regression analysis revealed risk recognition ability, attachment anxiety, state dissociation, and self-efficacy as significant predictors of revictimization. The final model accurately classified 82.4% of revictimized, 59.1% of victimized and 93.1% of non-victimized women. The overall classification rate was 80%.

**Conclusions:**

This study suggests that risk recognition ability, attachment anxiety, self-efficacy, and state dissociation play a key role in revictimization. Increased risk recognition ability after an interpersonal trauma may act as a protective factor against repeated victimization that revictimized individuals may lack. A lack of increased risk recognition ability in combination with higher attachment anxiety, lower self-efficacy, and higher state dissociation may increase the risk of revictimization.

## Introduction

The prevalence of re-exposure to trauma in later life among individuals who experienced victimization in childhood or adolescence is high. There is widespread empirical evidence that child sexual abuse significantly increases the risk of repeated victimization in adulthood [1]–[4]. Overall, the data indicate a two- to three-times higher risk of revictimization among victims of child sexual abuse [1], [2]. Physical maltreatment in childhood also increases the probability of revictimization [5]–[7]. Given a prevalence of child sexual and physical abuse in girls of 8 to 31% [8], revictimization is clearly a relevant societal problem. Victimization and repeated victimization are associated with posttraumatic stress disorder [9], dissociation [10], and interpersonal problems [11], as well as with substantial psychological strain. The public health costs resulting from repeated victimization are substantial [12].

### Variables associated with victimization and revictimization

Previous reviews have found several psychological variables to be associated with victimization and revictimization [2], [3], [13], [14], namely, deficits in risk recognition ability, self-efficacy, and assertiveness, as well as increased sensation seeking, dissociation, feelings of guilt and shame, and attachment anxiety.

#### Risk recognition

Risk recognition ability is the ability to sufficiently recognize danger cues (e.g., in social interactions) and to correctly identify dangerous situations. Various studies on situational risk recognition have found that victims of sexual abuse show lower risk recognition ability than do non-victims [15], [16]. However, other studies have found no support for the association between risk recognition and sexual victimization history [17], [18]. Some studies even suggest that emotional risk recognition in victimized individuals is above average [19]–[21]. These inconsistent findings may be due to the fact that most of the studies did not distinguish between victimized and revictimized individuals. Messman-Moore and Brown [22], who *did* differentiate between victimized and revictimized women, found higher risk recognition in victimized women and lower risk recognition in revictimized women than in non-victimized women. Likewise, Wilson et al. [23] found that revictimized individuals show lower risk recognition ability than do victims of a single assault. To date, risk recognition has been assessed in college or community samples only; no previous studies have examined risk recognition in a clinical context with severely impaired patients.

#### Further relevant variables

Isolated studies investigating the link between self-efficacy and revictimization have found evidence for a relationship between the two [24], [25]. For example, Lamoureux et al. [24] found that low self-efficacy acts as a mediating variable between child sexual abuse and high-risk sexual behavior, which can increase the risk of revictimization [26].

Assertiveness refers to the ability to stand up for one's rights—for example, to say “no” without feeling guilty. Low assertiveness has been associated with sexual victimization and revictimization [27]–[29]. Gidycz et al. [30] found that women's low assertiveness was predicted by experiences of child sexual abuse. Furthermore, women who were revictimized in adulthood showed significantly lower assertiveness than did non-victims [28].

Sensation seeking is significantly linked to a number of risk-taking behaviors [31], [32]. There is, for example, evidence of associations between sensation seeking and risky sexual behavior [33], which in turn predicts sexual revictimization [26].

There is widespread evidence for an association between victimization and dissociation, that is, a feeling of detachment from one's physical and emotional experience [34], [35]. Revictimization has also been associated with dissociation as well as with longer latencies of processing trauma-related stimuli [36]–[38]. However, Risser et al. [9] found no significant link between dissociation and repeated victimization in a follow-up period. All previous studies have assessed *trait* dissociation; research examining *state* dissociation during risky situations as a variable potentially contributing to revictimization is lacking.

Feelings of event-related guilt and shame are frequent consequences of victimization [39], [40]. Also guilt- and shame-proneness are associated with victimization [41], [42]. Event-related guilt and shame have also been associated with revictimization [43], [44]. In their longitudinal study, for example, Feiring et al. [44] found that shame and self-blame predict the experience of future dating aggression in individuals with past child sexual abuse. Research examining guilt- and shame-proneness in association with revictimization is lacking.

Attachment anxiety refers to an increased need for reassurance and fear of abandonment in relationships. Two recent studies on attachment anxiety have reported a positive correlation between child sexual abuse and attachment anxiety in adulthood [45], [46]. Moreover, a prospective study on predictors of revictimization [46] found that attachment anxiety plays an important role in later revictimization.

Identifying the impact of different variables on revictimization is essential for the development of efficient interventions and for the optimization of existing programs to protect survivors of violence from repeated victimization. In order to identify relevant risk factors for revictimization, it seems important to distinguish variables associated with victimization only from variables associated specifically with revictimization.

Most previous studies on revictimization have used selective samples, such as samples of college students. Few have examined clinical samples. This raises the question of whether or not the variables thus far associated with revictimization also apply to more severely impaired women who are at the highest risk of revictimization [1]. Furthermore, there has been great variability in previous definitions of victimization and revictimization. Different studies report different inclusion criteria for victimization and revictimization (e.g., from exhibitionism to severe rape), and they have failed to determine whether the incidents experienced met specific trauma criteria. Furthermore, two traumatic events committed by the same perpetrator are often counted as revictimization. We propose to use the term *victimization* when an event of interpersonal violence is experienced as *traumatic* according to the criteria of the Diagnostic and Statistical Manual of Mental Disorders IV (DSM-IV) [47]. We propose to use the term *revictimization* when at least two different traumatic events are experienced in two different periods of life and committed by different perpetrators.

### Study aim and hypotheses

The aim of the study was to identify variables that predict revictimization in a clinical sample. A specific aim was to provide insights into which variables are specifically altered in revictimized women relative to women who were victimized in childhood or adolescence only.

We hypothesized that the variables risk recognition, guilt, shame, attachment anxiety, sensation seeking, state dissociation, assertiveness, and self-efficacy would predict revictimization. We expected that revictimized individuals would show lower risk recognition ability than victimized or non-victimized individuals, and that individuals victimized in childhood/adolescence only would show higher risk recognition ability than revictimized individuals or non-victimized individuals. In addition, we expected revictimized individuals to show higher levels of guilt-proneness, shame-proneness, attachment anxiety, sensation seeking, and state dissociation than individuals victimized in childhood/adolescence only, as well as lower levels of assertiveness and self-efficacy.

## Materials and Methods

### Ethics statement

The authors assert that all procedures contributing to this work comply with the ethical standards of the relevant national and institutional committees on human experimentation and with the Helsinki Declaration of 1975, as revised in 2008. The study was approved by the Freie Universität Berlin's Internal Ethical Review Board.

### Sample and recruitment

The sample comprised 85 adult women between the ages of 21 and 64 years (*M* = 35.4; *SD* = 11.5); 22 (26%) in the victimized group (VIC), 34 (40%) in the revictimized group (REVIC), and 29 (34%) in the non-victimized comparison group (NON-VIC). Victimized and revictimized participants were inpatients recruited at the department of Psychiatry at Charité – University Medicine Berlin. Non-victimized controls were female undergraduate students at the Freie Universität Berlin and women recruited through announcements on the internet.

The inclusion criterion for the victimized group (VIC) was exposure to one or more incidents of interpersonal violence—i.e., sexual abuse or physical maltreatment—during childhood (age 0–14) only or during adolescence (age 14–18) only. Incidents of violence must have been experienced as traumatic events according to the criteria of the DSM-IV [47]. The inclusion criteria for the revictimized group (REVIC) were exposure to two or more incidents of interpersonal violence that were experienced as traumatic events according to the criteria of the DSM-IV, that were committed by different perpetrators, and that occurred in at least two different periods of life (i.e., childhood, adolescence, or adulthood). The inclusion criterion for the non-victimized comparison group was lack of exposure to traumatic events. Exclusion criteria for all three groups were lifetime psychotic disorder, substance dependence or abuse within the last six months, or acute suicidality.

### Measures

#### Risk recognition ability

To assess risk recognition ability, we developed stimulus material in German, based on the risk perception vignette of Marx and Gross [48]. Participants listened to an audiotaped vignette of a man and a woman engaged in conversation and sexual activity resulting in a sexual assault. The intensity of both the man's threats and the woman's refusals escalated over time (see Table 1). The vignette contained various risk factors for date rape described in the literature [49], including alcohol consumption, sexual comments, verbal persuasion, ignoring the woman's refusal, a degree of isolation, and verbal threats and physical pressure, also increasing over time.

**Table 1:** Excerpts from the vignette used in the risk recognition task.

Time in minutes from onset	Interaction
1 : 25	(m) “Lisa, you look super sexy in your dress tonight. Would you like to go outside with me for a while?”
	(w) “No, it's too cold outside, but I'd like to drink something, maybe another coke.”
3 : 55	(m) “Kiss me, Lisa.” (…)
	(w) “I like kissing you but don't touch my butt, that's too fast for me.”
	(m) “Sorry, but so close to you I just about lose control.”
4 : 42	(w) “Don't you listen, Felix, I don't want you to touch my breasts!” (louder)
	(m) “Shh, be quiet, don't let the others hear us.”
	(w) “Stop it, please!”
	(m) “OK, then I should go home and we should probably stop seeing each other.”
	(w) “Come on Felix, don't be upset.”
5 : 10	(m) “I know you want it, Lisa! Kiss me! It's so hard to control myself!”
	(w) “Stop it! Get your hands out of my pants. You know I don't like that!”
	(m) “Come on, just a little bit! Stop acting up, Lisa!”
5 : 33	(m) “Don't make me hurt you, Lisa!”
	(w) “I've told you I don't want any more! Take your hands off me!” (cries)
	(m) “Lie down!”

*Note.* (m) = man; (w) = woman.

Risk recognition ability was assessed by measuring response latency—that is, the length of time before participants pressed a button to indicate that they felt uncomfortable. Higher latencies indicate lower risk recognition ability. To keep the vignette's potential effects on acute dissociation constant and to prevent hesitation caused by curiosity about how the interaction would continue, participants were told that they would be able to listen to the end of the vignette after pressing the button.

The script developed by Marx and Gross [48] has been used and validated in previous studies [50]–[53]. To examine the validity of the German version, we presented the script of the vignette to 20 undergraduate female students and six experts prior to the study. Experts were postdoctoral clinical psychologists and therapists who had worked with trauma victims. Raters were asked to assess whether the scenario was realistic and whether the risk for victimization increased over time the longer the woman remained in the situation. Both questions were rated on a scale from 0 to 7 (ranging from *strongly disagree* to *strongly agree*). Raters strongly agreed that the scenario was realistic (*M* = 5.8; *SD* = 1.28) and that the risk for victimization increased over time (*M* = 6.4; *SD* = 0.82). The interaction was narrated by professional actors.

#### Psychopathology and traumatic events

Current major depression, posttraumatic stress disorder (PTSD), and borderline personality disorder (BPD) were assessed by means of the Structured Clinical Interviews for DSM-IV Axis I and II Disorders (SCID-I, SCID-II) [54], [55]. The semi-structured interviews used to make DSM-IV diagnoses show good test–retest reliability [56], [57]. Traumatic events were assessed using an adapted version of sections 1 and 2 of the Posttraumatic Diagnostic Scale (PDS) [58]. The PDS is a 49-item self-report measure containing four sections assessing all DSM-IV criteria for PTSD. With an alpha of 0.94, the German version of the PDS shows high internal consistency [59]. In order to assess all experienced events in terms of the DSM-IV trauma criteria, we conducted the PDS in interview format. Additionally, 10 traumatic events specific to child sexual abuse and maltreatment were added to the PDS checklist (e.g., ‘‘being forced to watch sexual activities” or “violence between parents”). These additional items were based on selected items from the Maltreatment and Abuse Chronology of Exposure scale (MACE; Teicher and Parigger, unpublished) and the trauma list of the Clinician-Administered PTSD Scale (CAPS) [60]. To assess revictimization, we obtained the following information for each experienced event: the victim's age at the time, how often the event was experienced, and who perpetrated it. In applying section 2 of the PDS, we did not only assess whether the *most upsetting* traumatic event met the DSM-IV trauma criteria; rather, we evaluated all traumatic events experienced in this respect.

#### Sensation seeking

Form V of the Sensation Seeking Scale (SSS-V) [32], [61] contains 40 items yielding four subscale scores (Thrill and Adventure Seeking, Disinhibition, Experience Seeking, and Boredom Susceptibility). Cronbach's alphas>.75 are reported [62].

#### Self-efficacy

The General Self-Efficacy Scale (GSE) [63] is a unidimensional 10-item self-report scale designed to assess a general sense of perceived self-efficacy. Cronbach's alphas are reported to range from.76 to.90 [64].

#### State dissociation

The Dissociation-Tension-Scale acute (Dissoziations-Spannungs-Skala; DSS-acute) [65] consists of 21 items tapping dissociation and one assessing aversive inner tension. The authors report a Cronbach's alpha of.94.

#### Guilt- and shame-proneness

The short form of the Test of Self-Conscious Affect (TOSCA-3) [66], [67] presents 11 everyday-life situations in which things have gone wrong. For each situation, participants rate how likely they would be to respond with guilt and shame. Tangney and Dearing [67] report Cronbach's alphas ranging from.70 to.88.

#### Assertiveness

The 30-item short form of the Trait Emotional Intelligence Questionnaire (TEIQue-SF) [68], [69] is a self-report measure assessing 15 distinct facets of emotional intelligence. We used the assertiveness subscale. Internal consistency (Cronbach's alpha) of the subscales has been reported to range from.71 to.92.

#### Attachment Anxiety

The German version of the Experiences in Close Relationships Inventory (ECR) [70], [71] is a 36-item self-report measure consisting of two subscales, measuring attachment anxiety and avoidance in close relationships. We used the anxiety subscale, for which Ehrenthal et al. [71] report a Cronbach's alpha of.91.

#### Crystallized intelligence

We used the Multiple Choice Vocabulary Test (Mehrfach-Wortschatz-Test; MWT-B) [72] to test verbal intelligence. In healthy adults, results on the MWT-B correlate well with global IQ [72].

### Procedure

On arrival, participants were given a full explanation of the study procedures. They then gave written informed consent to participate in the study. Subsequently, sociodemographic information (e.g., age, education) was gathered, diagnostic interviews (SCID-I, SCID-II, PDS) were conducted, and participants completed the questionnaire measures (GSE, SSS-V, TOSCA-3, TEIQUE-SF, ECR, MWT-B). Each participant was given the following instructions on the computer monitor before the risk recognition task:

Please listen carefully to the following interaction and try to put yourself in Lisa's position. I would like you to signal, by pressing the colored key on the keyboard in front of you, as soon as you feel uncomfortable with what is happening to Lisa. You will only need to push the key once. Please continue to listen to the situation until it is finished, even if you have pressed the key.

The experimental procedure was programmed using the computer program e-prime. Both the audio material and the instructions were thus presented in standardized form. Response latency was saved automatically. Immediately after listening to the vignette, participants filled in the Dissociation-Tension-Scale (DSS-acute). After completing all measures, participants were debriefed, given the opportunity to ask questions, and compensated.

### Data analysis

Data were analyzed using SPSS version 20 [73]. Chi-square and univariate analyses of variance (ANOVA) were conducted to test for baseline group differences in age, verbal IQ, educational level, psychopathology, number of victimizations during childhood/adolescence, and type of trauma. We then conducted a multiple logistic regression analysis to determine whether the variables risk recognition ability, self-efficacy, state dissociation, shame, guilt, assertiveness, sensation seeking, and attachment anxiety predicted membership of the three groups (VIC, REVIC, NON-VIC). Revictimization was used as the reference category. To reduce multicollinearity, we centered the independent variables before entering them in the analysis. A goodness-of-fit test was applied to assess the extent to which the model provided better fit than a null model with no predictors. In proportion to the number of independent variables used in our study, the sample size of the three groups was relatively small for a logistic regression. We therefore used bootstrapping to obtain a more robust estimate of the confidence intervals. The statistical significance of the association between group membership and the respective variables was determined using a 95% bootstrap confidence interval (CI). The bootstrap procedure is recommended for small data sets [74], and its confidence intervals are asymptotically more accurate and more correct as well as more robust than are standard confidence intervals [75], [76]. To counteract problems caused by complete separation—that is, when the outcome variable can be perfectly predicted by a set of variables [77]—we set the number of bootstrap samples to 10,000. In the multiple logistic regression analysis model, *p* values <0.05 were considered significant. The data are available at https://www.researchgate.net/profile/Estelle_Bockers.

## Results

### Baseline group differences

Univariate ANOVAs were calculated to test for baseline group differences in age, verbal IQ, and educational level. Chi-square tests were conducted to compare victimized and revictimized individuals with respect to diagnosis of depression, PTSD, and borderline personality disorder. No significant differences were found (see Table 2). Likewise, chi-square tests revealed that victimized and revictimized women did not differ in terms of the number of victimizations during childhood/adolescence, χ^2^(5) = 4.42, *p* = .490, or the type of trauma (i.e., sexual abuse only, physical abuse only, or both), χ^2^(2) = 3.55, *p* = .169.

**Table 2:** Sociodemographic characteristics and psychopathology of the sample.

Variables	REVIC (n = 34)	VIC (n = 22)	NON-VIC (n = 29)	Groups	
				F-ratio *p*	
Age, M *(SD)*	35.88 (8.56)	33.45 (11.39)	37.41 (14.54)	.90	.411
Educational level, M *(SD)* ^1^	11.10 (2.16)	11.00 (1.63)	11.69 (1.56)	.97	.382
Crystallized intelligence, M *(SD)* ^2^	27.94 (4.49)	28.00 (3.67)	29.60 (2.18)	2.15	.123
				χ^2^(1)	*p*
PTSD, in%	52.94	40.91	-	1.05	.306
Depression, in%	29.41	18.18	-	1.07	.301
Borderline personality disorder, in%	44.12	45.46	-	0.002	.962

*Note.* ^1^ = measured in number of years in school; ^2^ = measured with the MWT-B.

### Logistic regression analysis predicting revictimization

A multiple logistic regression analysis was conducted to predict group membership using risk recognition ability, self-efficacy, state dissociation, shame, guilt, assertiveness, sensation seeking, and attachment anxiety as predictors. A test of the final model against a constant model was significant, indicating that the set of variables included reliably predicted group membership, χ^2^(16) = 95.15, *p* <.001. The nonsignificant goodness-of-fit test indicated good model fit, χ^2^(152) = 165.96, *p* = .207. Nagelkerke's *R^2^* = .76 indicated that the predictive quality of the independent variables used was moderately strong. Overall, the predictors accurately classified 82.4% of the revictimized women (see Table 3 for an overview of the predicted probabilities).

**Table 3:** Findings of the logistic regression analysis: observed and predicted classifications.

	Predicted			
Observed	REVIC	VIC	NON-VIC	% Correct
REVIC	28	6	0	82.4
VIC	6	13	3	59.1
NON-VIC	1	1	27	93.1
Overall% correct	41.2	23.5	35.3	80.0

Table 4 reports the descriptive statistics of the independent variables across the groups. Statistical tests of the individual predictors showed that only risk recognition, attachment anxiety, self-efficacy, and state dissociation were significantly associated with group membership (see Table 5). As shown in Table 5, risk recognition and attachment anxiety significantly distinguished between victimized and revictimized individuals. State dissociation and self-efficacy significantly distinguished between revictimized individuals and non-victimized controls.

**Table 4:** Descriptive statistics: Means and standard deviations (in parentheses) of potential risk factors for revictimization across all three groups.

Variable	1: REVIC (n = 34)	2: VIC (n = 22)	3: NON-VIC (n = 29)
Risk recognition (sec)	197.24 (117.46)	131.83 (91.26)	235.97 (77.66)
Self-efficacy	20.42 (6.14)	21.04 (7.20)	31.60 (4.52)
Assertiveness	5.37 (2.78)	7.49 (3.65)	10.55 (3.41)
Guilt-proneness	49.20 (4.22)	47.17(6.25)	44.10 (4.53)
Shame-proneness	41.10 (7.36)	38.69 (9.76)	29.27 (8.42)
Sensation seeking	16.51 (7.94)	14.86 (8.70)	17.51(6.21)
State dissociation	2.71 (2.11)	2.11(1.86)	.25 (.29)
Attachment anxiety	4.81 (1.11)	3.88 (1.30)	2.29 (1.56)

**Table 5:** Multinominal logistic regression model predicting revictimization.

	B (SE)	Bootstrapped 95% CI		
		Lower	Upper	
VIC vs. REVIC				
Constant	−.07 (25.15)	−1.73		1.05
Risk recognition	−.01 (.26) **	−.03		−.002
Self-efficacy	.00 (.07)	−.21		.20
Assertiveness	.14 (6.55)	−.15		.73
Guilt-proneness	−.03 (1.26)	−.30		.22
Shame-proneness	−.02 (3.51)	−.28		.20
Sensation seeking	−.04 (2.74)	−.29		.09
State dissociation	.04 (15.33)	−.72		.89
Attachment anxiety	−.74 (47.91) *	−2.60		−.12
NON-VIC vs. REVIC				
Constant	−4.11 (2976.12) **	−1551.12		−2.17
Risk recognition	−.01 (12.67)	−2.34		1.31
Self-efficacy	.23 (156.99) **	.00		73.16
Assertiveness	.21 (220.91)	−14.76		70.20
Guilt-proneness	−.19 (163.86)	−48.02		4.62
Shame-proneness	.15 (149.25)	−1.83		45.54
Sensation seeking	−.05 (62.44)	−17.46		8.78
State dissociation	−2.99 (2179.55) **	−1013.83		−1.23
Attachment anxiety	−1.20 (406.52)	−154.14		15.47

*Note*. Multiple logistic regression was performed with 10,000 bootstrap replications; SE  =  Standard error; CI  =  Confidence interval; * *p* = <.05, ** *p* = <.01.

## Discussion

The aim of this study was to identify variables that predict revictimization. We were particularly interested in predictor variables that distinguished between the victimized group and the revictimized group. Our results showed that the set of variables we assessed accurately classified 82.4% of revictimized individuals and 93.1% of victimized individuals. Risk recognition ability, attachment anxiety, state dissociation, and self-efficacy were significant predictors of group membership. Risk recognition ability and attachment anxiety significantly distinguished revictimized women from women who had been victimized during childhood or adolescence only. Thus, these variables were specifically related to revictimization but not to victimization. State dissociation and self-efficacy significantly distinguished revictimized women from non-victimized women. In the following, we discuss these results in detail.

The two trauma groups (VIC and REVIC) did not differ in terms of psychopathology, number of victimizations during childhood/adolescence, or the type of trauma. These findings suggest that revictimization is neither specifically associated with disorders such as PTSD or borderline personality disorder, nor with the type and frequency of victimizations during childhood.

Our data showed that lower **risk recognition** ability, measured in terms of response latencies, distinguished between the victimized and the revictimized group, but not between the revictimized group and the non-victimized group. These findings indicate that risk recognition may not be impaired in revictimized individuals, but rather increased in victimized individuals. This interpretation is in line with the findings of the only previous study on revictimization and risk recognition that has differentiated between victimized and revictimized individuals in accordance with our definition of victimization and revictimization Messman-Moore and Brown [22]. The authors found that women with childhood victimization only identified threat cues significantly faster than did revictimized women or women without a history of victimization. They explained the former group's ability to quickly identify threat as a result of a sensitization to danger cues. This sensitization may serve as a buffer against future revictimization that may not be present in revictimized individuals. A lack of this potential buffer in combination with impairments in other variables in revictimized women may create a specific vulnerability for revictimization. The fact that revictimized and non-victimized individuals did not differ in terms of risk recognition ability suggests that additional variables are involved in revictimization. Thus, delayed or detrimental responses to real-life risky situations might be influenced not only by lower risk recognition, but also by other individual variables, such as self-efficacy and state dissociation, or by other, as yet unconsidered, variables that are impaired in victimized individuals.

**Attachment anxiety** also differentiated revictimized from victimized individuals in our study, with revictimized individuals showing higher levels of attachment anxiety. These findings are consistent with those of the only previous study that has assessed revictimization and attachment anxiety: Reese-Weber and Smith [46] identified attachment anxiety as an important predictor of revictimization. As Nurius [78] pointed out, from an individual perspective, there are always relative costs of both taking and not taking a specific action in a specific situation. Individuals with high levels of attachment anxiety are significantly more concerned about being rejected by others [79]. Thus, the individual cost of showing direct resistance towards an acquaintance in a risky situation may be much higher, which might increase the risk of revictimization. To our knowledge, the present study was the first to empirically assess attachment anxiety and revictimization in a clinical sample. Further research is therefore needed to corroborate our findings.

**Self-efficacy** differentiated revictimized individuals from non-victimized individuals; lower self-efficacy significantly predicted revictimization. This finding is in line with the results of studies indicating that impaired self-efficacy is associated with risky sexual behavior [24], [25] and with later victimization in a population of adolescent females [24], [25]. However, in the present study, lower self-efficacy did not significantly differentiate between revictimized and victimized individuals. Hence, it cannot be concluded that low self-efficacy is specifically associated with revictimization. Rather, low self-efficacy may be associated with victimization in general, rather than with revictimization in particular. In addition, the present study examined *general* self-efficacy. Further research into the role of *situation-specific* self-efficacy (e.g., *sexual* self-efficacy) seems warranted, particularly with regard to different forms of revictimization.

Finally, higher **state dissociation** positively predicted the likelihood of revictimization. Like self-efficacy, state dissociation differentiated between revictimized and non-victimized, but not between revictimized and victimized individuals. This pattern of results suggests that state dissociation is related to revictimization, but it may also be related to victimization in general. These findings are consistent with the results of a prospective study by Iverson et al. [36]–[38], who found higher levels of dissociation to be associated with a higher revictimization risk. Our finding of higher state dissociation as a predictor of revictimization is also in line with the theoretical work of Chu [80], who suggested that victimized individuals are at a particularly high risk of revictimization during acute numbing, when normal anticipatory anxiety is unavailable. In contrast to our finding, Risser et al. [9] found no significant link between dissociation and repeated victimization in a follow-up period. However, these authors assessed a college population. Additionally, to our best knowledge, the present study was the first to empirically assess *state* dissociation, rather than *trait* dissociation and revictimization; further research is therefore needed.

Contrary to our expectations, none of the other variables (sensation seeking, assertiveness, guilt, or shame) were found to predict revictimization. In the longitudinal study by Feiring et al. [81], shame and self-blame did emerge to be positive predictors of revictimization. In contrast to the present study, however, these authors assessed abuse-specific feelings of shame and self-blame. General shame and guilt-proneness may be more strongly associated with victimization than with revictimization. Another possible explanation for the lacking association between revictimization and guilt and shame may be the potentially higher level of alexithymia in individuals after traumatization. Alexithymia—that is, difficulty identifying and describing one's own feelings [82]—was not examined and controlled in the present study. Shame and guilt were assessed using an explicit self-report measure. In future research, we propose that guilt and shame be assessed by means of implicit measures. The lack of significant results with respect to assertiveness is surprising given that the highest level of assertiveness was found in the non-victimized group, whereas the lowest level of assertiveness was found in the revictimized group. This may be explained by the inclusion of overlapping constructs in the logistic regression model, such as self-efficacy.

Previous research found that risky sexual behavior predicts sexual revictimization [26]. For that reason, we assumed that sensation seeking, which is linked to risky sexual behavior [33], may be associated with revictimization. However, our results showed that sensation seeking did not predict revictimization. The general concept of sensation seeking including dimensions like *Thrill and Adventure Seeking* or *Experience Seeking* relates to behaviors which produce feelings of excitement but are not necessarily risky in an interpersonal context. Persons with high levels of sensation seeking may have different underlying motives for their behavior than victims of child sexual abuse showing risky sexual behavior or engaging in relationships with violent partners. Whereas high sensation seeking may be due to a higher individual need for novelty [83], sexually risky behavior in victims of interpersonal violence may be due to higher attachment anxiety and the fear of abandonment [84].

Generally, the discrepancy between these and previous findings may also be due to differences in the samples. Previous studies on revictimization have assessed college and community samples, rather than clinical samples [27]–[29].

Some strengths and limitations of the study warrant consideration. To our knowledge, this study is the first to examine the association of a set of variables including risk recognition, attachment anxiety, self-efficacy, and state dissociation with revictimization in an inpatient clinical population. One potential limitation is the relatively small sample size, which reduces the statistical power to detect small effects. However, our calculations indicate that there was adequate power to detect effects of moderate size. Furthermore, although risk recognition was measured implicitly, the risk recognition task cannot represent risk scenarios completely realistically. For example, patterns of physical and emotional arousal and state dissociation probably differ in real-life dangerous situations. Participants may also have reacted in a socially desirable manner, which could have led them to indicate that they felt uncomfortable earlier than they would have done if facing the same situation in reality. Because most of the victimized and revictimized women in our sample knew their perpetrators, we presented an acquaintance risk scenario with someone the fictional woman had dated once. However, many of the revictimized women in our study reported victimizations in long-term relationships, which were not represented in our risk scenario. Furthermore, although this paradigm has been used and validated in several previous studies [50]–[53], the German version of the risk recognition task needs further validation. The findings of the pilot study we conducted prior to the present study showed good validity.

Despite these limitations, and although the study design does not allow causal relationships to be drawn, we believe the findings of our study to be of importance as they highlight the relevance of four variables predicting revictimization: risk recognition, attachment anxiety, self-efficacy, and state dissociation. Particularly risk recognition and attachment anxiety seem to be important variables increasing the risk of revictimization. Our findings underline the importance of risk recognition training and interventions focusing on attachment anxiety to prevent later revictimization in victimized women. Recent studies have shown that attachment insecurity and attachment anxiety, often believed to persist throughout the lifespan, are also significantly changeable through psychotherapeutic interventions such as psychodynamic interpersonal psychotherapy [85], [86].

In sum, our findings suggest that lower risk recognition ability in victimized individuals in combination with higher attachment anxiety, higher state dissociation, and lower self-efficacy may further increase the risk of revictimization.
